# Human B Lymphomas Reveal Their Secrets Through Genetic Mouse Models

**DOI:** 10.3389/fimmu.2021.683597

**Published:** 2021-07-16

**Authors:** Noushin Mossadegh-Keller, Gabriel Brisou, Alicia Beyou, Bertrand Nadel, Sandrine Roulland

**Affiliations:** ^1^ Aix Marseille Univ, CNRS, INSERM, CIML, Marseille, France; ^2^ Department of Hematology, Institut Paoli-Calmettes, Marseille, France

**Keywords:** germinal center (GC), follicular lymphoma (FL), diffuse large B cell lymphoma (DLBCL), genetically engineered mouse (GEMs), epigenetic modifier mutations

## Abstract

Lymphomas are cancers deriving from lymphocytes, arising preferentially in secondary lymphoid organs, and represent the 6th cancer worldwide and the most frequent blood cancer. The majority of B cell Non-Hodgkin lymphomas (B-NHL) develop from germinal center (GC) experienced mature B cells. GCs are transient structures that form in lymphoid organs in response to antigen exposure of naive B cells, and where B cell receptor (BCR) affinity maturation occurs to promote B cell differentiation into memory B and plasma cells producing high-affinity antibodies. Genomic instability associated with the somatic hypermutation (SHM) and class-switch recombination (CSR) processes during GC transit enhance susceptibility to malignant transformation. Most B cell differentiation steps in the GC are at the origin of frequent B cell malignant entities, namely Follicular Lymphoma (FL) and GCB diffuse large B cell lymphomas (GCB-DLBCL). Over the past decade, large sequencing efforts have provided a great boost in the identification of candidate oncogenes and tumor suppressors involved in FL and DLBCL oncogenesis. Mouse models have been instrumental to accurately mimic *in vivo* lymphoma-specific mutations and interrogate their normal function in the GC context and their oncogenic function leading to lymphoma onset. The limited access of biopsies during the initiating steps of the disease, the cellular and (epi)genetic heterogeneity of individual tumors across and within patients linked to perturbed dynamics of GC ecosystems make the development of genetically engineered mouse models crucial to decipher lymphomagenesis and disease progression and eventually to test the effects of novel targeted therapies. In this review, we provide an overview of some of the important genetically engineered mouse models that have been developed to recapitulate lymphoma-associated (epi)genetic alterations of two frequent GC-derived lymphoma entities: FL and GCB-DLCBL and describe how those mouse models have improved our knowledge of the molecular processes supporting GC B cell transformation.

## Introduction

**Figure 1:**
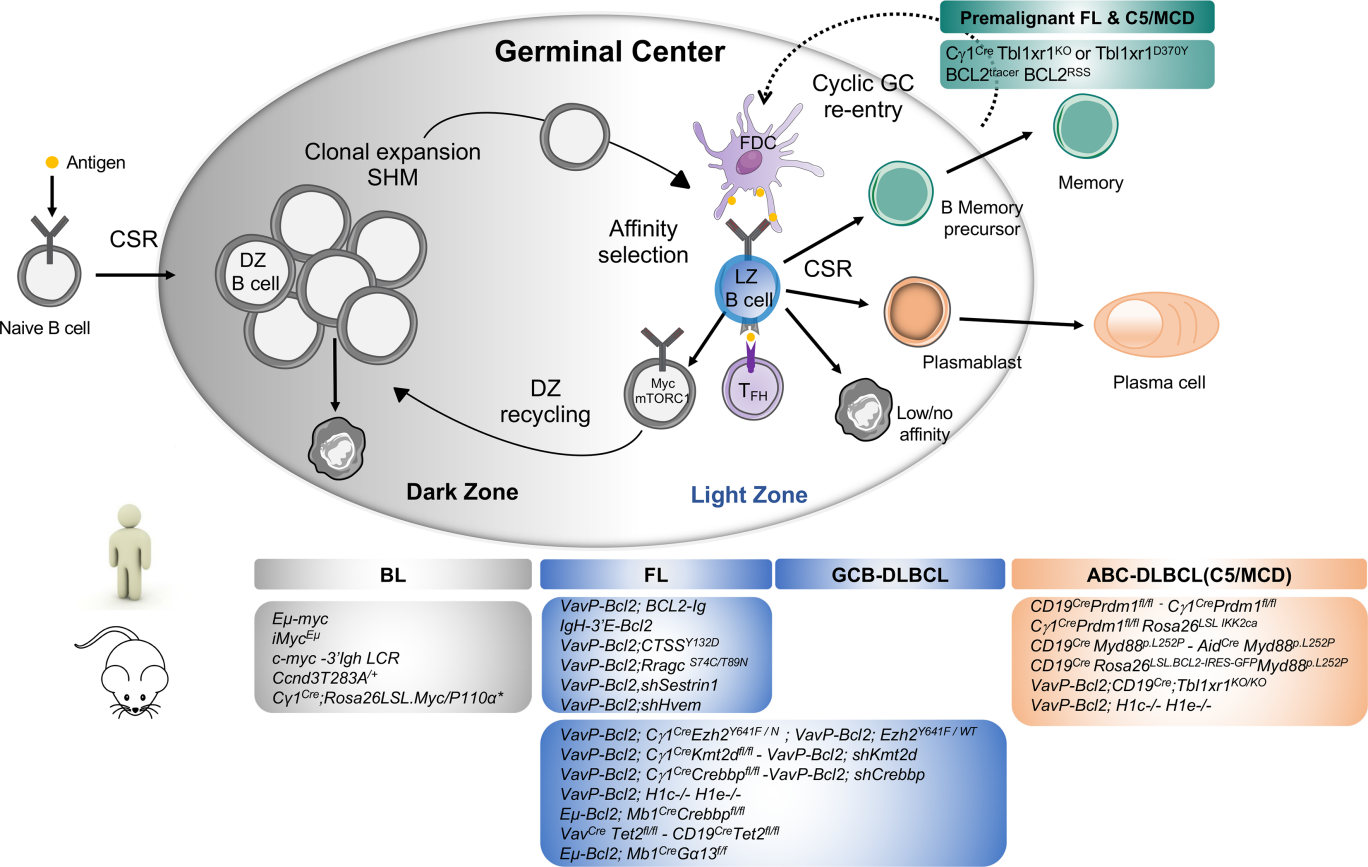
Major mouse models of human B cell lymphomas linked to their putative normal B cell counterpart. Top Panel: Schematic representation of Germinal Center B cells. Activated B cells enter in the GC dark zone (DZ), the site of clonal expansion and somatic hypermutation. Only a subset of DZ B cells will be selected to pass in the GC light zone (LZ) while most DZ B cells undergo apoptosis. GC LZ is the site of affinity-based selection where high-affinity B cells tend to capture more antigen from FDC and receive more T cell help through CD40/CD40L interaction driving their re-entry into the DZ for defined rounds of proliferation and SHM. In the LZ, owing to the failure to receive T cell help and acquisition of damaging BCR mutations, most GC B cells will undergo apoptosis while some LZ B cells that gained productive BCR mutations and enhanced affinity will be selected and terminally differentiate into memory B or plasma cells depending on the strength of T cell help they received [recently reviewed in (5)]. In normal immune response, current models suggest that memory B cells tend to exit early the GC response following low level of T cell help and typically display less SHM and reduced levels of affinity maturation than plasma cells. A limited number of memory B cells can re-enter into secondary GC upon antigenic recall for additional rounds of mutations (6, 7). Cyclic memory cell reactivation of mutated B cells into the GC is however a driving mechanism leading to B cell transformation (8, 9). Bottom panel: The most important genetically-engineered mouse models are linked to the human lymphoma they intend to mimic distinguishing Burkitt Lymphoma (BL) arising from transformation of DZ cells, Follicular Lymphoma and GCB-DLBCL from LZ cells and ABC-DLBCL arising from Activated B/plasmablastic or memory B cells. BL is characterized by Myc translocations between the immunoglobulin heavy or light chain locus. Transgenic mice engineered to dysregulate MYC expression under proximal or distal enhancers Eµ-*Myc* (10), iMyc^Eµ^ (11) and *c-myc*3’LCR (12) led to the development of aggressive lymphomas with Burkitt-like phenotype with high penetrance and short latency *in vivo*. Conditional overexpression of Myc and PI3K signaling (Cγ1^Cre/wt^;Rosa26^LSL.Myc/P110α*^) in the GC cooperate to drive BL-like tumors identifying PI3K pathway activation as a key survival element in Myc-driven lymphomas (13). Modeling Cyclin D3 ^T283A^ gain-of-function mutations in B cells—which are recurrent event in DLBCL and sporadic BL (14, 15)—leads to increased DZ proliferation and occasional lymphoproliferative disease in older mice highlighting the need for additional events to exert its oncogenic function (16, 17). ABC-DLBCL are typically characterized by an enhanced activity of the NF-κB survival pathway and the co-occurrence of genomic aberrations in BCR (CD79B), MYD88, TBL1XR1, 18q gains affecting BCL2 and PRDM1 inactivation interfering with normal plasma cell differentiation, all defining features of the C5/MCD genetic subtype (18–20). Single conditional knockout of *Prdm1* in B cells or specifically in the GC reveal lymphoma lesions of post-GC origin (Bcl6^−^, Irf4^+^) indicative of a preplasmablastic stage in only 20% of animals and with a long latency (18, 21). Constitutive activation of NF-κB pathway with *Prdm1* disruption in the GC cooperate to drive DLBCL-like tumor development resembling human ABC-DLBCL (22, 23). Conditional expression in B cells of an oncogenic Myd88^L252P^ allele plus BCL2 overexpression (mimicking BCL2 copy number gains) result in the development of aggressive post-GC lymphomas recapitulating many genotypic, transcriptomic and signaling features of ABC-DLBCL pathogenesis (24, 25) notably the formation of the My-T-BCR (Myd88/TLR9/BCR) supercomplex driving NF-κB mediating survival signals (26) and detection of autoreactive antibodies suggesting a role for self-antigens in driving BCR stimulation as previously proposed in human and mouse models (27, 28). Somatic mutations in *TBL1XR1* are enriched in the MCD/C5 genetic subtype (18). Conditional deletion of *Tbl1xr1* or expression of TBL1XR1^D370Y^ mutant allele in B cells generates aberrant memory B cells which are more prone to cyclic re-entry into GC reaction thereby providing additional evidence on how skewed GC/Memory B cell dynamics act as a major pathogenic mechanism in lymphoma development (8, 9, 29). Combined with Bcl2 overexpression, *Tbl1xr1* mutant mice ultimately give rise to canonical post-GC extranodal ABC-like lymphomas with a proportion of B cells manifesting with a memory B cell phenotype consistent with a putative memory B cell origin of ABC-DLBCL tumors (21).

The germinal center (GC) is a specialized immune structure localized in secondary lymphoid organs—including lymph nodes, tonsils, and spleen—that forms upon antigenic challenge to support the B cell receptor (BCR) affinity maturation process. In this transient, highly dynamic structures, activated B cells undergo clonal expansion, somatic hypermutation (SHM) of immunoglobulin (Ig) variable genes, selection and eventual differentiation into memory B cells or long-lived plasma cells (PC) (1). The GC is canonically divided into two principal zones: the dark zone (DZ), where B cells undergo clonal expansion and accumulate SHM upon activation-induced-cytidine deaminase (AID) responsible of BCR diversification, and the light zone (LZ), where GC B cell will test their newly acquired mutated Ig for improved affinity to antigen through interaction with immune complex-coated follicular dendritic cells (FDCs) and selection by a limited number of CD4+ T follicular helper cells (T_FH_) residing in the LZ (2). Within the LZ, B cells can have several fates: (i) a small subset of high-affinity GC B cells, selected in the LZ, will recycle back in the DZ to undergo further cycles of expansion/mutation/selection (3, 4), (ii) some selected LZ B cells can directly exit the GC differentiating into effectors such a memory B cells or plasma cells and (iii) LZ cells with low/no affinity BCRs following SHM due to a lack of antigen engagement and subsequent T cell help die by apoptosis (**Figure 1**). In the GC LZ, the strength and intensity of the signal received by B cells from T_FH_ cells, which is largely influenced by BCR affinity, mainly determines B cell fate. Recently, the dynamic transcriptional changes characterizing the GC cycle between LZ and DZ have been further refined through single cell gene expression approaches revealing a continuum of cell states between LZ and DZ and highly orchestrated group of molecular programs that co-evolve during the GC response (30–34).

T dependent humoral response proceeds in several steps triggered by multiple finely orchestrated cellular interactions that affect B cell response through the activation and repression of specific transcriptional programs. Molecular control of this highly dynamic process is complex and involves several transcriptional regulators such as transcription factors and epigenetic regulators that are frequently targeted by somatic mutations driving lymphomagenesis. After antigen encounter and T cell co-stimulation, B cells get activated through BCR, CD40 and toll like receptor (TLR) signalling, inducing NF-κB activation and the expression of genes involved in B cell activation and proliferation driving GC initiation (35, 36). BCL6 is a transcriptional repressor which play a central role in GC initiation and maintenance (37). Its expression is triggered by B–T interaction during the early initiation of the GC response, where it allows B cells to migrate into the center of B cell follicles through the downregulation of EBI2 and S1PR1 and induction of CXCR4. Once GCs are established, BCL6 coordinates the GC response by repressing thousands of genes involved in different cellular processes (T cell mediated B cell activation, BCR/CD40 signaling, apoptosis, DNA damage response, cellular cycle checkpoints,…) (38, 39). In this way, BCL6 allows DZ cells to establish a hyper-proliferative program while tolerating DNA damage caused by SHM without triggering proliferation arrest or apoptosis. In addition, BCL6 prevents signal transduction from several membrane receptors, thus preventing B cells from premature differentiation. BCL6 expression must be repressed to allow B cells to exit the GC. Two signals cooperate to repress BCL6: BCR activation *via* Ag presented by FDCs and CD40 activation *via* CD40L expressed by T_FH_ cells (38, 39). The transcription factor cMYC is also essential for GC initiation. Indeed, about 2 h after B cell activation, GC precursors transiently express c-MYC before expressing BCL6 and co-express c-MYC and BCL6 for a short period of time, allowing the initial proliferation phase leading to GC formation. Once the GC is established, c-MYC is then partially repressed by BCL6 (3, 4). Back in the LZ, high-affinity B cells that take up the Ag integrate signals from the BCR and additional signals through several receptors, including CD40, BAFF and TLRs which ultimately activate NF-κB. NF-κB signaling and CD40 costimulation result in cMYC re-expression in selected B cells that return to the DZ for further rounds of cell division (40). On the other hand, this activates IRF4 which, when highly expressed, represses BCL6 expression, thus promoting GC programme silencing and post-GC differentiation (35, 41). Indeed, at high concentration, IRF4 induces BLIMP1 expression which allows plasma cell differentiation (42). Memory B cell differentiation process is less understood but is thought to derive from B cells with low affinity BCR receiving a weak signal from T_FH_ cells, two transcription factors have been involved in this process: BACH2 and more recently HHEX (43, 44).

B cell lymphomas are cancers that develop from the malignant transformation of B lymphocytes at different stages of ontogeny (45). From naive to memory and plasma cells, most differentiation steps are associated with a malignant B cell counterpart defined historically as the cell-of-origin on the basis of histological definitions, phenotype and resemblance of transcriptomic profiles (46). Rapid clonal expansion, genomic instability, tolerance to DNA damage and metabolic reprogramming are physiological GC B cell-specific features that makes them permeable to lymphomagenesis (47, 48). Accordingly, GC B cells are considered at the origin of frequent B cell lymphomas—namely follicular lymphoma (FL), GC B cell-diffuse large B cell lymphoma (GCB-DLBCL), and Burkitt lymphoma (BL) (**Figure 1**). FL have a follicular growth pattern reminding normal GC architecture, with the presence of T_FH_ cells and FDC stromal cells, carries heavily mutated Ig genes known to occur in the GC primarily and retain a closely related signature to LZ B cells (49, 50). GCB-DLBCL is more transcriptionally reminiscent of LZ B cells while BL is more similar to DZ B cells (46, 51). Of note, single-cell gene expression analyses of mouse (16, 30, 44, 52) and human GC B cells (31–33) have revealed that GC B cell transcriptional states span a continuum from LZ to DZ, and that a large proportion (between 30 and 50%) of GC B cells are in an intermediate state between the two zones. Besides providing important datasets to understand GC transcriptional programs during the normal immune response, these studies offered a more granular survey of human GC B cells states that can serve as references for re-assessing and revisiting the concept of GC B cell lymphoma cell-of-origin (31, 32).

Yet, gene expression profiling only accounts for a portion of GC B cell lymphoma heterogeneity and the advent of next generation sequencing (NGS) has provided a great boost in the identification of genetic alterations (mutations, translocations, copy number alterations…) involved in FL and DLBCL oncogenesis, and has revealed an increased complexity of the lymphoma genetic landscape (14, 18, 19, 53–55). Although GCB-DLBCL and FL represent two clinically and histopathological distinct lymphoma entities, it has become apparent that these two subtypes are far more intricated from a genetic point of view as illustrated by shared multiple recurrent genetic lesions such as BCL2 alterations, mutations in epigenetic regulators (*KMT2D*, *CREBBP*, *EZH2*, *EP300…*) and immune receptor signaling genes suggesting at least clonal evolution from a similar precursor cell and shared oncogenic pathways (53, 56). Accordingly, the recently defined EZB/C3 genetic subtype is composed by a majority of tumors with a GC B cell gene expression profile and is enriched for the most common genetic abnormalities such as BCL2 translocations and EZH2 mutations.

Among others, mouse models of lymphoid malignancies have advanced our understanding of lymphomagenesis [reviewed in (57–60)] and currently support the biological investigations on the most common putative driver mutations alone or in combination. The limited access to (pre)malignant biopsies during the initiating stage of lymphoma development, the difficulties to recapitulate in *in vitro* experimental systems the complexity of the GC reaction during an immune response (61), the spatial and (epi)genetic heterogeneity across and within human lymphomas make the development of genetically engineered mice models the most suitable tool i) to characterize the molecular mechanisms by which candidate lymphoma mutations contribute *in vivo* to lymphomagenesis either alone or in combination and ii) to trace how tumours grow and evolve over time by recapitulating the precise timing at which the genetic lesions happens in human settings iii) to test the effects of targeted pharmacological agents and iv) the synergy between co-occurring genetic alterations. In this review, we will present recent insights on FL and GCB-DLBCL lymphoma mouse models in which genetic alterations targeting the epigenome, immune signaling or metabolic pathways have been accurately recapitulated and for which mechanistic studies yielded new insights on how GC regulatory programs are hijacked by somatic mutations to prevent the resolution of ‘pseudo-tumoral’ GC B cell features and facilitate lymphomagenesis (**Table 1**). Starting with the founder BCL2 translocation common to the pathogenesis of most FL and GCB-DLBCL, we will focus on how genetically engineered mice (GEM) of (epi)genetic alterations shed new lights on the link between B ‘cell-intrinsic’ lesions and their cell-extrinsic functions to drive lymphoma development by promoting the remodeling of an aberrant immune niche and contributing to immune surveillance mechanisms.

**Table 1:** Relevant mouse models of follicular lymphoma and germinal center B cell diffuse large B cell lymphomas.

Target gene	Mouse Model	Model type	Target cells	Mutation type	Latency (mo)	Disease phenotype	References
***Bcl2***	*BCL2-Ig*	Transgenic (*BCL2-Ig* minigene)	B cells	Overexpression	18	Hyperplasia, FL	(62)
	*Eµ-BCL2*	Transgenic (driven by 5’ Igh *Eµ* enhancer)	B cells	Overexpression	18	LPD	(63, 64)
	*VavP-Bcl2*	Transgenic (driven by *VavP* promoter)	All hematopoietic	Overexpression	10–18	FL	(65)
	*IgH-3’E-Bcl2*	Knock-in (driven by Igh *3’RR* enhancers)	Mature B	Overexpression	7–14	FL	(66)
	*huBcl2^RSS^*	Transgenic (Inactive human *BCL2* locus flanked by RAG recombination sequence signals, RSS)	pre-B	Sporadic overexpression		no tumors	(8)
	*huBcl2^RSS^;AID^Cre^ R26^LSL.YFP^*	BM chimera: HSPCs from AID^Cre^Rosa26^LSL.YFP^ transduced with *huBcl2^RSS^* tracer	pre-B (BCL2) GC for EYFP	Sporadic overexpression	10	*In situ* Follicular Neoplasia	(8)
***Crebbp;Bcl2***	*Eµ-Bcl2;Mb1^Cre^Crebbp^fl/fl^*	Conditional knockout combined with *Bcl2* overexpression	pro-B	Loss of function	13	FL, GCB-DLBCL	(67)
	*VavP-Bcl2;C*γ*1^Cre^Crebbp^fl/fl^*	Conditional knockout combined with *Bcl2* overexpression	GC	Loss of function	18	FL, GCB-DLBCL	(68)
	*VavP-Bcl2;shCrebbp*	BM chimera of HSPCs from *VavP-Bcl2* transduced with *shCrebbp*	All hematopoietic	Loss of function	2	FL, GCB-DLBCL	(69)
***Kmt2d;Bcl2***	*VavP-Bcl2;C*γ*1^Cre^Kmt2d^fl/fl^*	Conditional knockout combined with *Bcl2* overexpression	GC	Loss of function	13	FL, GCB-DLBCL	(70)
	*VavP-Bcl2;shKmt2d*	BM chimera: HSPCs from *VavP-Bcl2* transduced with *shKmt2d*	All hematopoietic	Loss of function	5	FL, GCB-DLBCL	(71)
**Ezh2**	*Cd19^Cre^Ezh2^Y641F/+^*	Conditional knockin (endogenous promoter)	Pre-B	Gain of function	12	DLBCL	(72)
***Ezh2;Bcl2***	*VavP-Bcl2;Ezh2^Y641F/WT^*	BM chimera: HSPCs from *VavP-Bcl2* transduced with *Ezh2^Y641F^*	All Hematopoietic	Gain of function	3–4	GCB-DLBCL	(73, 74)
	*Bcl2hi; Cd19^Cre^Ezh2^Y641F^*	BM chimera: HSPCs from Cd19CreEzh2Y641F transduced with Bcl2	Pre-B	Gain of function	7	FL, GCB-DLBCL	(72)
	*VavP-Bcl2;C*γ*1^Cre^Ezh2^Y641F^*	Conditional knockin combined with *Bcl2* overexpression	GC	Gain of function	–	GCB-DLBCL	(74)
***Ezh2;Bcl6***	*C*γ*1^Cre^Ezh2^Y641F/+;^ IµBcl6*	Conditional knockin combined with *Bcl6* overexpression	GC	Gain of function	6–12	GCB-DLBCL	(75)
***H1c/H1e***	*VavP-Bcl2;H1c^-/-^ H1e^-/-^*	Non-conditional knockout with *Bcl2* overexpression	All hematopoietic	Loss of function		DLBCL	(76)
***Tet1***	*Tet1^KO/KO^*	Non-conditional knockout	All hematopoietic	Loss of function	12	LPD, DLBCL	(77)
***Tet2***	*CD19^Cre^ Tet2^f/f^*	Conditional knockout	Pre-B	Loss of function	4–18	CLL	(78)
	*Vav^Cre^Tet2^f/f^*	Conditional knockout	All hematopoietic	Loss of function	_	GCB-DLBCL	(79)
**Hvem**	*VavP-Bcl2;shHvem*	BM chimera: HSPCs from *VavP-Bcl2* transduced with *shHvem*	All hematopoietic	Loss of function	4	FL	(80)
**Ctss**	*Vav-Bcl2; CTSS^Y132^*	BM chimera: HSPCs from *VavP-Bcl2* transduced with mutated human *CTSS^Y132D^*	All hematopoietic	Gain of function	2	FL	(81)
	*Vav-Bcl2; CTSS^HIGH^*	BM chimera: HSPCs from *VavP-Bcl2* transduced with overexpressed *CTSS*	All hematopoietic	Overexpression	2	FL	(81)
***Gα13***	*Mb1^Cre^Gα13^f/f^*	Conditional knockout	pro-B	Loss of function	>12	GCB-DLBCL	(82)
***Gα13;Bcl2***	*Eµ-Bcl2;Mb1^Cre^Gα13^f/f^*	Conditional knockout combined with *Bcl2* overexpression	pro-B	Loss of function	10	GCB-DLBCL	(82)
***RragC^mut^***	*VavP-Bcl2;RragC ^S74C/T89N^*	Knock-in mice crossed with *VavP-Bcl2*	All hematopoietic	Gain of function	10	FL	(83)
***Sestrin1***	*VavP-Bcl2,shSestrin1*	BM chimera: HSPCs from *VavP-Bcl2* transduced with sh*Sestrin1*	All hematopoietic	Loss of function	–	FL	(84)

## Modeling t(14;18) Translocation and Bcl2 Overexpression

FL represents an attractive model to study the mechanisms by which lymphoid B cells undergo neoplastic initiation and progression in mouse models (50). While FL is considered as a disseminated GC-derived B cell neoplasia, acquisition of the t(14;18) translocation—that lays the *BCL2* gene under the transcriptional control of Ig heavy chain (*IGH*) regulatory regions—constitutes a critical early, likely primary, event in the natural history of the disease occurring in bone marrow (BM) pre B cells during illegitimate V(D)J recombination. It is now well established that t(14;18), although present in 85% of FL and about 30% of GCB-DLBCL patients, is not enough to transform B lymphocytes as t(14;18)-positive circulating B cells are detectable at low frequency (one in a million lymphocytes) in up to 70% of healthy adults who never develop the disease (85), indicating that complementary “hits” must further accumulate, presumably during the later phases of GC B cell maturation (86). Attempts to model the t(14;18) translocation and Bcl2 overexpression have started in the late 80’s with first generation of Bcl2 transgenic mouse models (62).

### Eµ-*BCL2* and *BCL2*-Ig

In 1989, to mimic the human *BCL2-IGH* translocated allele and assess the tumorigenic potential of BCL2 *in vivo*, Mc Donnell and colleagues developed the first *BCL2* transgenic mice bearing a human *BCL2*-Ig minigene where expression of BCL2 is restricted to B cells (62). The mice developed follicular hyperplasia made of small naive B cells—expressing markers such as B220, IgD, IgM and Igκ—with prolonged survival *in vitro*. In original studies, after 18 months of age these mice showed high-grade lymphomas although at a low penetrance and interestingly half of those mice harbored *c-myc* rearrangement. More recent follow-up studies showed that 40% of *BCL2*-Ig mice develop FL-like tumors expressing GC markers (PNA^+^BCL6^+^) by 17–18 months when chronically immunized with sheep red blood cells (SRBC) over 6 months (87). Strasser and colleagues similarly engineered a transgenic strain Eµ*-BCL2*, where *BCL2* is placed under the control of the 5’ *IGH* enhancer Eµ (63). This model also showed an expansion of small B cells and plasma cells but did not yield tumor development, however an increased incidence of other B-lymphoid neoplasms was observed (64).

### VavP-*BCL2* and IgH-3’E-*Bcl2*

The first experimental model that faithfully reproduced the human disease in term of localization, histology, phenotypic and genotypic features involved expression of Bcl2 in all hematopoietic cells. Egle and colleagues generated the VavP-*Bcl2* model where the *Bcl2* transgene is controlled by *Vav* gene regulatory sequences, which confer Bcl2 expression in multiple hematopoietic lineages (65). The mice developed, in 15–25% of cases, isotype-switched, somatically mutated Ig and disseminated lymphomas at 10 months of age. In contrast to Eµ*-BCL2* transgenic models of the same age, VavP-*Bcl2* mice develop spontaneous expansion of PNA^+^ GC lesions in otherwise ‘healthy’ mice, a premalignant condition which is strongly dependent on CD4 T cell help as *in vivo* removal of CD4 cells almost abolished GC hyperplasia. In this model, CD4 T cells make a critical input into the exaggerated GC reactions and eventually the onset of FL. A second model developed by the group of Boxer (66) consisted of a IgH-3’E-*Bcl2* knock-in mice, where the *IgH* 3’ enhancer regions was integrated 3’ of *Bcl2* locus, thereby mimicking the effects of the long distance *IgH 3’* enhancer on Bcl2 expression and limiting Bcl2 expression to mature B cells. In addition to an altered B-cell differentiation and increased B cell numbers in the spleen, lymph node (LN) and BM, these mice recapitulated, between 7- and 14-months, typical histopathological features of GC-experienced FL-like tumors expressing the GC markers PNA and Bcl6 surrounded by FDC networks and CD4^+^ T cells. Despite its main limitations due to the unspecific Bcl2 deregulated expression in all hematopoietic cells, VavP-*Bcl2* is currently one of the most popular models to study *in vivo* the role of secondary genetic alterations that are frequently found in combination with BCL2 in FL and GCB-DLBCL genetically engineered mouse models.

These studies support the notion that despite a survival advantage conferred to B cells by BCL2, the lymphomagenic process requires additional hits (genetic and/or immunological) for transformation.

### Modeling the Early Steps of FL Development: *BCL2^tracer^* Model

Although several BCL2-engineered models have provided the initial proof-of-principle that BCL2 ectopic expression leads to FL and high-grade lymphomas, the expression of BCL2 in all B cells (and all T cells for some models) do not represent a true premalignant intermediate stage seen during human lymphomagenesis, where the first t(14;18) event occur in a single B cell in the BM, and is carried on until its ectopic expression in the GC. Instead, pan-B cells BCL2 mouse models generate a polyclonal hyperplasia of naive B cells which is not a known progression step in human FL pathogenesis and as a consequence does not allow studying the early steps of clonal emergence and disease progression. The *BCL2^tracer^* mouse model has been engineered to mimic “sporadic” t(14;18) translocation (8). This transgenic model relies on the introduction of potent RAG recombination sites at the vicinity of an inactivated human BCL2 transgenic minilocus. In this construct, recombination allows to turn on ectopic BCL2 expression in only few B cells, and at the appropriate window of B cell development in the BM pre-B cells. The recombination breakpoints provide unique PCR-based clonotypic markers to study early steps of clonal emergence and expansion in mouse blood/tissues at different time points, allowing a precise analysis of clonal progression kinetics. As expected, the emergence of BCL2^+^ B cells was traced in various tissues at low frequency (1 in 10^5^ to 10^6^) recapitulating the “healthy human t(14;18) carrier” situation. At steady state or after acute immunization with a T-cell dependent antigen, BCL2 alone was not able to drive progression of BCL2-expressing cells into a tumor after 18 months of follow-up, confirming that lymphomagenesis is a stepwise process where premalignant B cells require the accumulation of secondary (epi)genetic alterations to progress into a tumor. However, a chronic immunization protocol with SRBC accelerated genomic instability by allowing BCL2-overexpressing B cells to give rise to memory cells that preferentially underwent iterative rounds of GC entry, allowing multiple rounds of AID-mediated mutagenesis over time to ultimately form premalignant *in situ* FL structures, the earliest known intermediate preceding human FL. Although this model alone does not form FL lesions, it has been instrumental to propose a revised model of early lymphomagenesis whereby cyclic reactivation of BCL2^+^ memory B cells within new GC reactions would constitute a major pathogenic mechanism facilitating clonal expansion and accumulation of secondary mutations in FL precursors. It is likely that this reactivation in humans operates over decades before clinical manifestation (that we will never reach during the mouse lifespan) and ultimately contribute to the generation of a heterogeneous population of aberrant memory B cell intermediates resembling clonal FL evolving in asymptomatic patients years before diagnosis. Interestingly, this model also led to identify an “immunological 2^nd^ hit” in FL, departing from the all-genetic, cell-intrinsic concept of lymphomagenesis.

## Modeling Epigenetic Alterations

Recurrent mutations affecting histone modifying enzymes are a hallmark of GC-derived lymphomas, more particularly in FL. Within DLBCLs, these mutations are enriched within the GCB-DLBCL subtype, with further enrichment within the newly described EZB/C3 genetic subtype. Inactivating mutations of the H3K4 lysine methyltransferase *KMT2D* are found in 70 to 90% of FL cases and up to 30% of DLBCL cases representing the most frequently FL mutated genes in these lymphomas after t(14;18) translocations. Approximately 50 to 70% of FLs and ∼25% of DLBCLs carry acquire inactivating mutations in *CREBBP*, whereas its paralog *EP300* is mutated in ∼5% of cases. Activating mutations of the H3K27 histone methyltransferase *EZH2*, a component of the polycomb repressive complex 2, are found in 10 to 25% of FL cases and 20% of GCB-DLBCL cases (14, 18, 19, 53–55, 88–90). Overall, 95% of FL patients manifest with at least one chromatin modifier gene mutation. Thorough genomic inference analyses of the clonal evolution patterns in sequential pairs of FL at diagnosis *vs.* relapse/transformation showed that recurrent inactivating mutations in *CREBBP* and *KMT2D* represent early events in FL evolution and are likely to be present in the CPC pool supporting a founder role for these events.

### *CREBBP* and *EP300* Inactivation

CREBBP and EP300 are highly homologous histone acetyltransferases (HAT) that modify gene expression through H3K27 acetylation at enhancer domains of both histone and non-histone substrates. In 50 to 75% of cases, mutations of *CREBBP* are missense inactivation of the acetyltransferase catalytic domain, the remaining mutant alleles causing truncation or loss of expression (54, 55, 69, 90). *CREBBP* and *EP300* mutations are usually detected only at one allele and exclusive fashion, which is thought to be explained by the compensatory and redundant role of these enzymes. The precise timing and location where *CREBBP* or *EP300* mutations happens during B cell transformation is still unclear in humans, but there is large agreement from clonal evolution studies and in premalignant FL conditions that CREBBP is an early event (91). Several groups have therefore attempted to investigate the role of hemizygous *vs.* homozygous *Crebbp* inactivation on mature B cell differentiation and lymphoma development *in vivo* using different strategies from early inactivation in all hematopoietic cells, early B-cell specific deletion or GC-specific deletion [C*γ*1-Cre (92)], in combination or not with BCL2 transgenic models.

Horton and colleagues studied the consequences of *Crebbp* deletion in the hematopoietic stem cell compartment thanks to a pIpC-mediated Mx1-Cre recombinase system (93). Those animals developed B cell lymphoproliferative disorders accounting for 29% of all deaths. The B cell lesions localized mostly in the spleen and blood and stained with B220, CD19 and surface IgM indicating their mature B cell origin, however these tumors did not express GC markers. The occurrence of these lymphoproliferative disorders was preceded by the accumulation of lymphoid progenitors characterized by a hyperproliferative state and an altered DNA damage response linked to the loss of p53 activation in the absence of *Crebbp*. Interestingly, the authors found no enrichment for *Bcl6* targets among Crebbp binding sites in their system suggesting different epigenetic changes when *Crebbp* is deleted in hematopoietic stem cells (HSC). This data suggests that *CREBBP* mutations acquired early during hematopoiesis may contribute to the emergence of B cell lymphomas although this model might not be relevant for FL or DLBCL progression as the tumors do not exhibit GC features. It also indicates that the timing of mutation acquisition has an important impact on B cell development. In humans, detection of CREBBP mutations in HSCs remains a very rare event (93).

Three others groups investigated the B-cell deletion of *Crebbp* in combination with BCL2 overexpression to fit with the frequent co-occurrence of the two alterations in human FL and DLBCL (67–69). Jiang et al. recapitulated *Crebbp* downregulation and Bcl2 overexpression *in vivo* using a shRNA retroviral infection system of VavP*-Bcl2* hematopoietic stem progenitor cells (HSPCs) transplanted into lethally irradiated wild-type recipient mice. They observed an acceleration of lymphoma onset in double-mutant vs. *VavP-Bcl2* mice. Lymphoma cells expressed B220, CD19 and IgM and were characterized by somatically mutated Ig locus confirming their GC B cell origin. Importantly, a similar phenotype was observed with a shRNA targeting *Ep300*. The second model developed by Zhang and colleagues consisted in cohorts of Cγ1-Cre (92) and Cd19-Cre *Crebbp^flox^* ^/^ *^flox^* and *Crebbp^flox^* ^/^ *^+^* animals where inactivation of *Crebbp* was induced in GC or developing B cells respectively. After a follow-up of 18 months they did not observe any significant difference between *Crebbp* mutant mice and their littermate controls concluding that loss of *Crebbp* alone at early or late stages of B cell differentiation was not sufficient to induce lymphomagenesis. The generation of mice crossing Cγ1-Cre *Crebbp^flox^* ^/^ *^flox^* mice with VavP*-Bcl2* transgenic mice led to a significant increase in the incidence of B cell malignancies resembling human FL (92% in double mutant versus 61.5% in VavP*-Bcl2* controls). Furthermore, the tumors were characterized by a follicular architecture, were largely of GC origin with Bcl6 expression and presence of mutated Ig genes. In an extension of this study and to investigate in parallel how CREBBP and EP300 contribute to normal GC B cell physiology, Meyer et al. established mouse lines carrying single “floxed” HAT genes that are excised only in activated B cells in the GC (Cγ1-Cre) (94). In accordance with previous studies (95), Meyer et al. confirmed that loss of *Crebbp* in GC B cells led to increased GC formation while *Ep300* loss led to an opposite effect with decreased proportion of GC B cells in immunized mice (94). Transcriptomic analyses of purified GC B cells obtained from these two strains revealed that the set of genes modified by *Ep300* loss or *Crebbp* loss was different with expression of DZ transcripts preferentially repressed in *Ep300*-deleted GC B cells while LZ genes were preferentially decreased in *Crebbp*-deleted GC B cells providing a mechanistic explanation for the reduced numbers of GC B cells observed in the GC-deficient *Ep300* model. The most interesting phenotype comes from the drastic reduction of GC B cells observed when both HAT genes are deleted from the same GC cells. Indeed, the GC response was completely abrogated indicating that the ability of GC B cells to proliferate and differentiate relies on the combined activity of both acetyltransferases likely due to their overlapping and partially redundant functions. Interestingly, *CREBBP*-mutated lymphoma B cells maintained this dependency toward EP300 enzymatic activity which identify a unique vulnerability that provide exciting opportunities of targeting single mutant CREBBP or EP300 GC-derived lymphomas.

In a third model, Garcia-Ramirez et al. produced strains of Mb1-Cre *Crebbp^flox/+^* or *Crebbp^flox/flox^* mice where *Crebbp* was deleted at early pro-B cell stage in the BM. *Crebbp* deletion at early stages of B cell development led to reduced frequencies of B-cell subsets with reduced numbers of total B220^+^ B cells in BM and spleen. When crossed with the Eµ*-BCL2* mouse model, these animals showed higher frequencies of GC B cells in the spleens after SRBC immunization and occasionally develop clonal B cell lymphoma with low penetrance and long latency (13 months). Histology was similar to human FL grade 3 or DLBCL and tumoral cells expressed Pax5 and Bcl6 and displayed SHMs consistent with a GC B cell origin (67).

Overall, these three independent *in vivo* models confirmed an oncogenic cooperation between *CREBBP* loss of functions and BCL2 overexpression to promote lymphomagenesis *in vivo*. Mechanistically, these studies showed that CREBBP, and likely EP300, maintain H3K27 acetylation at certain enhancer that are poised during GC reaction and whose reactivation is required for GC exit and terminal differentiation. This includes genes involved in immune synapse, downstream effectors of BCR and NF-κB or terminal differentiation (*Irf4*, *Nfkb2*, *Cd40*) and antigen presentation, the most notable being MHC class II molecules which are critical for B cell terminal differentiation. In normal GC cells, CREBBP targeted enhancers are direct targets of BCL6 and transiently repressed by BCL6/SMRT/HDAC3 complexes that deacetylate H3K27. Upon selection signals received in the LZ allowing GC exit, these enhancers recover H3K27ac state as CREBBP can directly acetylate BCL6 to inactivate its function by preventing the interaction with co-repressor complexes. By impairing the reactivation of these enhancers leaving unopposed BCL6 oncogenic activity, CREBBP loss of function disrupt the expression of immune synapse genes and their downstream signaling pathways, resulting in accumulation of aberrant GC B cells that fail to properly respond to exit signals from the GC microenvironment thereby promoting lymphoma progression. In human FL, decreased MHC II expression and reduced CD4 and CD8 T cell infiltrations have been described in *CREBBP*-mutant FL. A similar association between *CREBBP* inactivation and reduced expression of MHC class II is observed in murine lymphoma models which alters mutant GC cells ability to present antigen to CD4^+^ cells. Interestingly, *CREBBP*-mutant lymphomas become dependent to HDAC3, the histone deacetylase opposing the effect of CREBBP, that has been identified as a relevant therapeutic target in these tumors (69). Mondello et al. recently showed that HDAC3-selective inhibitors have a dual effect by reversing CREBBP-mutant aberrant epigenetic programming limiting lymphoma growth inhibition while restoring antitumor immunity, notably antigen presenting-genes (96).

### *KMT2D* Loss of Function

KMT2D is a component of the COMPASS complex involved in transcriptional activation through H3K4 monomethylation of gene enhancers in B cells. The majority of *KMT2D* mutations are nonsense events leading to a truncated protein lacking the enzymatic SET domain involved in H3K4 methylation resulting in a loss of function (90). To study the consequences of *Kmt2d* inactivation during B cell differentiation, Zhang and colleagues generated a conditional *Kmt2d* knock-out model relying on a Cre-Lox system with conditional deletion of B cells early during B cell development (Cd19-Cre *Kmt2d^flox/flox^*) or after GC initiation (Cγ1-Cre *Kmt2d* ^flox/flox^). Early deletion of *Kmt2d* lead to a higher number and enlarged GC formation while this effect was not seen with late GC deletion and *in vitro*, *Kmt2d*-deficient cells displayed a proliferative advantage compared to wild-type cells. However, *Kmt2d* deficiency in B cells alone upon early or late inactivation was not sufficient to induce FL or DLBCL *in vivo*. Kmt2d protein was mainly found on putative GC enhancers and global H3K4 methylation levels were diminished in *Kmt2d* mutant mice. Moreover, although GC-specific deletion was insufficient to initiate malignant transformation, *Kmt2d^ko^*-VavP*-Bcl2* double-mutant mice developed B-cell lymphoproliferative disorders with an incidence of 78% (44% for VavP*-Bcl2* alone*)*, with tumors expressing *BCL6* and *PAX5* consistent with a GC origin and recapitulating a spectrum of histopathological features ranging from early FL to DLBCL (40% early FL, 31% FL and 27% DLBCL) (70).

Using a different experimental system, Ortega-Molina and colleagues also explored cooperation between bcl2 and *Kmt2d* deletion in B cell lymphomagenesis. Using a retroviral infection system of shRNAs transduction to silence *Kmt2d* in HSPCs from VavP*-Bcl2* donor mice following transplantation into irradiated wild-type mice, an acceleration of the lymphomagenesis process as well as an increase in the incidence of FL-like tumors (from 30 to 60%) was observed in double-mutant mice compared to shRNA control vectors, validating the tumor suppressive role of *Kmt2d* in B lymphocytes. At preclinical stages of the disease, Ortega Molina et al. showed that after immunization, the number of GC B cells was increased when *Kmt2d* was suppressed. Moreover, GCs persisted for a longer period than in control mice. K*mt2d* loss was associated with a decrease in IgG1 production suggesting a dysfunction of the class switch recombination processes (71). Interestingly, the generation of *Kmt2d^flox/flox^*Cd19-Cre crossed with a strain overexpressing AID led to the development of aggressive lymphomas resembling DLBCLs and confirms that, independently from BCL2 expression, genetic instability linked to AID overexpression cooperates with *Kmt2d* loss to promote lymphomagenesis.

Integrative genomic analyses from human samples carrying *KMT2D* mutations and *Kmt2d*-mouse FLs showed that genes differentially expressed in *Kmt2d*-mutated lymphomas were mostly repressed and affected a set of genes involved in terminal differentiation programs and GC exit, such as CD40 and BCR signaling, regulation of apoptosis, control of cell migration and proliferation. *KMT2D* mutations result in persistent demethylation of enhancers and failure of the respective genes to respond to signals, notably CD40 signaling from T_FH_. Moreover, absence of *Kmt2d* affects negatively the expression of major B-cell tumor suppressors such as *Tnfaip3*, *Socs3*, *Tnfrsf14*, *Asxl1* or *Arid1a*. In conclusion, lack of *Kmt2d* leads to an aberrant repression of key genes normally required for GC exit, favoring an abnormal GC B cell outgrowth and failure to differentiate leading eventually to lymphoma development. Of note, these studies showed once more that the stage and/or the timing of a given (epi)genetic alteration has a strong influence on the transcriptional changes occurring in the GC. The developmental stage at which *Kmt2d* mutations are introduced in human precursor tumor cells is still unknown but it has been hypothesized that epigenetic reprogramming may require multiple rounds of cell divisions to allow the replacement of modified histones by non-modified histones explaining why *Kmt2d* inactivation after GC initiation may have a more modest phenotype than early inactivation.

### *EZH2* Gain of Function

EZH2 is a H3K27 methyltransferase part of the Polycomb Repressive complex-2 (PRC2). Heterozygous gain of function mutations, preferentially affecting the EZH2 SET domain at the Y641 residue and making EZH2 more efficient at H3K27 trimethylation (97), are found in up to 30% of GCB-DLBCL and FL and *de facto* enriched in EZB/C3 DLBCL subtype (18, 89, 98). Several groups have investigated the functional role of wild-type and mutant EZH2 during GC reaction and B cell lymphomagenesis (73, 75, 99, 100). Using a Cγ1-Cre *Ezh2^flox/flox^* strain, Béguelin et al. observed a marked reduction in GC B cells after immunization. They reproduced this phenotype in immunized wild-type mice treated with an EZH2 inhibitor targeting wild-type and mutant *EZH2*, establishing that EZH2 is required for GC formation (73). Similar observations were made by Caganova et al. underlining that under normal conditions, EZH2 enables GC formation at least in part by suppressing cell-cycle checkpoint genes like CDKN1A, impairing DNA damage responses to support centroblast proliferation and silencing essential plasma cell differentiation genes, particularly *Blimp1* and *Irf4* (99, 100).

To understand how *EZH2^Y641^* hotspot mutation perturbs GC development and drives lymphomagenesis, Béguelin and colleagues developed two mouse strains conditionally expressing the mutant *Ezh2^Y641N^* or *Ezh2^Y641F^* in GC B cells upon Cγ1-Cre recombinase (73, 75). Upon immunization, both models caused oversized GCs and displayed increased abundance of H3K27me3 mark at critical GC B cell bivalent promoter leading to permanent silencing of EZH2 target genes. The GC phenotype appears to be mediated through cooperative and mutually interdependent actions of EZH2 together with the transcriptional repressor BCL6 and BCOR repressive complex. These *Ezh2* mutant mice did not develop lymphomas. However, early activation (CD19-Cre) of mutant *Ezh2* in an independent study led to aggressive DLBCL in about 12 months (72). VavP*-Bcl2* HSPCs were transduced with retroviruses expressing *Ezh2^Y641F^*, *Ezh2* ^WT^ or an empty vector and transplanted into lethally irradiated recipient mice subjected to SRBC immunization every 4 weeks to induce GC formation. *Ezh2^Y641F^ Bcl2* ^+^ chimeric mice led to early lymphoma development in 70% of mice at 111 days (*vs* 20% in mice overexpressing *Ezh2* ^WT^ *Bcl2* ^+^ and none in the *Bcl2* control at that stage), characterized by enlarged spleen and liver and resembling morphologically to DLBCL with centroblastic morphology (73, 74). These data demonstrate that *EZH2* gain of function mutations accelerate GC B cell lymphomagenesis in cooperation with Bcl2 overexpression, recapitulating features of GCB-DLBCL. Similar results were obtained when transgenic and knock-in *Ezh2* strains engineered to express heterozygous mutant Ezh2 in GC B cells were crossed with VavP*-Bcl2*. Importantly, homozygous expression of mutant *Ezh2* phenocopies the *Ezh2* knock-out phenotype further attesting the requirement for the maintenance of the wild-type allele for *Ezh2* mutant enzymatic activity.

Recent studies questioned whether EZH2 mutation has additional and qualitatively distinct function in lymphomagenesis beyond simply being a more potent version of the wild-type enzyme. Along these lines, Ennishi et al. identified a strong enrichment of *EZH2* mutations in human DLBCL cases with loss of MHC-I and MHC-II expression linked to a reduced number of tumor-infiltrating lymphocytes and less T cell cytolytic activity (74). To investigate in detail the consequences of EZH2 mutations on MHC expression and immune microenvironment, the authors relied on two different mouse models. First, they used the *Ezh2*-mutant model developed by Béguelin et al. where VavP*-Bcl2* hematopoietic progenitors are infected with *Ezh2*-mutant containing retrovirus before re-injection into lethally irradiated recipient mice. Second, they used Cγ1-Cre *Ezh2^Y641N^* or *Ezh2^Y641F^* mice crossed with VavP*-Bcl2* strain which develops DLBCL-like tumors. In both experimental systems, MHC-I and MHC-II expression were significantly reduced in mutant *Ezh2* mice compared to wild-type mice, with reduced infiltration of CD3, CD4 and CD8 T cells in the tumor microenvironment, establishing *Ezh2* gain-of-function mutation as a driver of MHC downregulation in GC lymphomagenesis, and eventually favoring immune escape. Importantly they also showed that EZH2 epigenetic switch-off of MHC molecules, driven by transcriptional repression of MHC-I/II transactivators, could be reversed with EZH2 inhibitors. The ability to restore MHC expression provides an interesting proof of concept in combining epigenetic reprogramming small molecules with immunotherapeutic approaches.

More recently, Melnick and colleagues further strengthened the concept that *Ezh2* mutation initiate GC derived lymphomagenesis by escaping immune effector recognition and inducing a remodeling of the GC immunological niche (101). Using competitive BM chimera to track the cellular dynamics of *Ezh2^Y641F^* B cells during GC reaction, they found that *Ezh2* mutants manifested a competitive growth advantage in the GC. This competitive advantage led to GC hyperplasia characterized by an increase of a cycling LZ cell population, without maturation blockade, associated with the expansion of FDC network. Using droplet based single cell transcriptomics and advanced histone mass spectrometry technologies, they assessed how *Ezh2* mutation affected GC B cell cellular and histone methylation dynamics in an unbiased manner. They found that the epigenetic reprogramming imprinted by *Ezh2* mutation through the reinforcement of the repressive program induced by H3K27me3 accumulation led to the abolition of LZ B cells’ dependence toward T_FH_ emanating signals. Indeed, DZ re-entry of *Ezh2*-mutated centrocytes was clearly diminished and these GC B cell escaped T_FH_-mediated clonal selection. The ability of *Ezh2*-mutant GC B cells to proliferate within the LZ was linked to FDC interaction as this ability was impaired when FDC function was abolished through injection of soluble lymphotoxin receptor β. Remarkably, this study revealed how activating mutation of *EZH2* induces a premalignant FL-like niche allowing B cells to persist as slowly proliferative centrocytes without T_FH_ help and in a FDC dependent manner.

The above studies provide important insights on an emerging paradigm where one of the most critical function of epigenetic modifier mutations in promoting GC B cell transformation goes beyond the sole reprogramming of the GC epigenome, but instead arise from failure of GC exit signals to restore expression of genes that normally regulate immune signaling pathways and antigen presentation (47). Of therapeutic interest for the targeting of the FL common precursor cells, this remodeling of the immune synapse at least with *EZH2* mutations tends to occur early during lymphomagenesis.

### Linker Histone Loss of Function

Linker histones (H1) are additional chromatin modifying genes involved in the organization and stabilization of the nucleosome structure, supporting the folding of chromatin into higher-order conformation, and regulating its epigenetic state through the recruitment of histone modifiers. Heterozygous H1 mutations, found in up to 44% of FL and 27% of GCB-DLBCL, are mostly missense events affecting the globular C-terminal domain which led to the loss of protein function with impaired chromatin binding (53). Among them, H1C and H1E are the most common affected H1 isoforms observed in B cell lymphomas (102, 103).

To investigate the functionality of these isoforms in lymphomagenesis, Yusufova and colleagues used the previously described H1c^−/−^ H1e^−/−^ mice model (104). At early time points after chronic SRBC immunization, those animals manifested lymphoproliferative disease with invasiveness of B220^+^ B cells in extranodal tissues such as liver and lungs. Further analysis using competitive BM chimera revealed a competitive advantage for H1c/H1e-deficient B cells characterized by a specific increase of cycling LZ B cell population expressing Gl7, Fas and cd86 markers whereas no effect was found in other mature and immature B cell populations. Notably, H1 deficiency enables a chromatin decompaction in GC B cells with an enrichment in stem cell genes that become desilenced in H1-deficient GC cells. Given the frequent co-occurrence of *H1C* and *H1E* mutated genes with BCL2 overexpression in lymphomas, they crossed H1c^−/−^ H1e^−/−^ mice with *VavP-Bcl2* mice. Loss of H1 isoforms caused a more extensive disruption of lymph node architecture with diffuse infiltration of immunoblastic cells, along with an extensive invasion of B220^+^ B cells and CD3^+^ T cells in extranodal tissues establishing H1 proteins as *bona fide* tumor suppressors. RNA sequencing analysis of lymphoma-like tumors in mouse and humans revealed a significant enrichment for stem cell signature and serial transplantations confirms that loss of H1 conferred lymphoma cells with enhanced self-renewal potential. These findings enlighten the contribution of H1 linker deletion in driving malignant transformation where epigenetic marks changes favor a relaxed state chromatin in GC B cells, increasing B cell fitness advantage by allowing self-renewal proprieties and may expose DNA to further AID-mediated additional hits.

### TET1 and TET2 Loss

Besides chromatin modifiers genes, a number of studies have demonstrated methylation and disruption of cytosine methylation [5-methylcytosine (5mC)] patterning as another factor linked to the biology of B cell lymphoid malignancies (105). The methyl-cytosine dioxygenase *TET2* (ten-eleven translocation 2) missense or truncated mutation is present in 6–12% of GCB-DLBCL (14). *TET2* mutations are known to occur early in human HSCs and can be found in individuals with clonal haematopoiesis. Whether early *TET2* mutations has a driving role in DLBCL was explored in multiple models. 5mC is well established as an epigenetic mark associated with transcriptional silencing, notably of tumor suppressor genes. TET2 is involved in active DNA demethylation, catalysing the oxidation of 5mC to 5-hydroxymethylcytosine (5hmC). Recently, it has been appreciated that 5hmC also functions as an epigenetic mark, and when linked to gene enhancers, is associated with activation of nearby genes.

Conditional deletion of *Tet2* specifically in the B cell compartment with CD19-Cre Tet2*^fl/fl^* mice showed B cell transformation mimicking chronic lymphocytic leukemia (78). Programmed deletion of *Tet2* in hematopoietic cells (Vav-Cre) or B cells (CD19-Cre) in immunized animals disrupt the ability of GC B cells to undergo CSR and terminal differentiation. Furthermore, conditional deletion of *Tet2* at the GC stage results in a preneoplastic GC hyperplasia, blockage of GC exit and PC differentiation evolving in DLBCL-like tumors, confirming its role as a *bona fide* B cell tumor suppressor (79). Mechanistically, this phenotype is due to the focal loss of 5hmC at enhancers linked to B cell differentiation. Indeed, *Tet2^−/−^* GC B cells feature disruption of many enhancers linked to GC exit signaling pathways, antigen presentation, and terminal differentiation genes. This mechanism is conceptually similar to the functions of the histone modifiers in DLBCL which fails to restore the immune synapse. Interestingly, *TET2* and *CREBBP* mutations are mutually exclusive in DLBCL (106), thus a combined mouse model could be engineered to find a potential therapeutic vulnerability in DLBCL.

The methyl-cytosine dioxygenase *Tet1* (ten-eleven translocation 1) is also an important regulator of 5-hydroxymethylcytoine and interestingly transcriptionally silenced in FL. Cimmino and colleagues engineered *Tet1-*deficient mice where B cell lymphoma development was promoted resulting in a diminished survival compared to wild type mice (77). *Tet1*-deficient mice exhibited lymphadenopathy and hepato-splenomegaly. Splenic tumors were characterized by a massive infiltration of proliferating lymphocytes disrupting the normal architecture and expressing the GC markers Bcl6 and Irf4 but not the PC marker Cd138. When combined with Bcl2 overexpression, Tet1-deficient B cell lymphomagenesis was accelerated up to 10 weeks post-transplantation. Altogether, deletion of *Tet1* and *Tet2* in mice induces phenotypically predominant DLBCL tumors supporting a suppressor role in mature B cells.

## Modeling Evasion From Immune Surveillance and Dissemination

The influence of the GC microenvironment on B cell development which provides essential signals for the survival, selection and differentiation is well established with several actors residing in the GC LZ such as T follicular helper cells (T_FH_), follicular dendritic cells (FDC), regulatory T cells (Treg), macrophages and stromal cells (19). FL is the paradigm of a B cell malignancy strongly dependent on direct interaction with a GC-like permissive microenvironment that co-evolves with malignant cell clones as a part of a dynamic interplay (49). Recent studies in both FL patients and genetically-engineered mouse models have started to highlight the link between B ‘cell-intrinsic’ tumor genetic alterations and their cell-extrinsic functions during lymphomagenesis by contributing to escape of immune surveillance mechanisms. We will discuss the latest studies showing how the *TNFRSF14 or CTSS* frequent alterations in FL modify the TME/malignant B cells crosstalk and contribute to lymphoma development either by affecting antigen processing and hiding from the immune system, or modifying its composition to become tumor-supportive.

### *Tnfrsf14* Loss in FL

*TNFRSF14*, the gene encoding the HVEM receptor located on 1p chromosome is one of the most frequent cell surface protein, deleted or mutated in >40% of FL cases and enriched in GCB-DLBCL (14, 18, 19). HVEM is expressed at the surface of B cells as well as other cell types and has multiple ligands including LIGHT or BTLA. Besides its role as a signaling receptor, HVEM can act as ligand and transmit signals into BTLA-expressing cells notably T_FH_ (107). How loss of *HVEM* contributes to lymphomagenesis has been the focus of two independent and complementary *in vivo* studies (80, 108). The first study took advantage of the well characterized VavP*-Bcl2* model that recapitulates key aspects of the genetics and pathology of human FLs to generate BM chimeras where knockdown of *Hvem* was mediated by transduction of shHvem into VavP-*Bcl2* HSCs followed by reconstitution of irradiated wild-type hosts. Knockdown of *Hvem* in all hematopoietic system caused a significant acceleration and increased penetrance of lymphoma development compared to VavP*-Bcl2 controls* with 90% of animals carrying tumors at 100 days. Despite the absence of B-cell specificity of the shRNA transduction strategy, only B cells were enriched with the short-hairpin construct indicating the *Hvem* knockdown in T cells was unlikely to participate to the lymphomagenic effect. Mechanistically, besides cell-autonomous activation of B cells, *Hvem* loss and the consequent loss of interaction with Btla triggers the amplification of T_FH_ producing high amounts of TNF and Lymphotoxin, the two non-redundant factors involved in lymphoid stromal cell differentiation and maintenance, and favors lymphoid stromal cell activation including FDC and FRC (Follicular Reticular Cells). This seminal study offers the first demonstration of a functional impact of a B-cell specific genetic alteration on the polarization of a FL-supportive microenvironment (80). Of therapeutic interest, immunotherapeutic delivery of a soluble HVEM receptor, *via* modified CAR-T cells, inhibited the growth of lymphoma by restoring the BTLA-HVEM interaction highlighting the essential role of the tumor/microenvironment dialog in lymphomagenesis. In an independent study, Mintz et al. reported that in models of *Bcl2* overexpression in B cells, *Btla* deficiency in T cells led to a similar GC B cell outgrowth and accelerated lymphomagenesis than *Hvem* deficiency in B cells proposing an alternative mechanism by which the Btla-Hvem axis functions as an cell-extrinsic suppressor in lymphomagenesis (108). Using a chimeric mouse system, they identified that during a normal immune response, *Hvem* restrains B cell proliferation, differentiation and selection by reducing the delivery of signal in *trans* through the Btla-Hvem axis on T_FH_ cells. *Hvem* mutation in B cells would lead to a loss of negative signaling in T_FH_ cells and allows *Hvem*-mutant B cells to receive exaggerated helper signals that promote proliferation and accrual of AID-mediated mutations. Collectively, those data provide important evidence for a cell-extrinsic tumor suppressor role of *Hvem*. The ways in which increased signaling *via* CD40 and other T cell–derived helper factors cooperates with Bcl2-overexpression in lymphoma development remain to be fully elucidated.

### *CTSS* Alterations in FL

Cathepsin S (CTSS) is part of a family of cysteine proteases whose role is essential in the regulation of normal immune response through its activity on antigen processing, B cell expansion and communication with CD4^+^ T cells. By cleaving the CD74 chaperone protein bound on MHC class II molecules, CTSS enzymatic activity results in a smaller peptide CLIP that will be displaced and allow variable antigenic peptides to bond to MHCII and present at the cell surface. Recurrent hotspot mutations and gene amplifications of *CTSS* have been recently described in 6 and 13% of FL patients respectively (81, 109) and mechanistically, CTSS^Y132D^ hotspot mutation promotes activation of the protein and increases its protease activity. To assess how the most common CTSS^Y132D^ mutations or CTSS overexpression contribute to accelerate lymphoma development, Dheilly et al. generated a chimera mouse model of FL using the VavP*-Bcl2* mice as HSPC donor cells and expressing either mutated human CTSS or overexpressing human CTSS. Both models revealed an oncogenic role of CTSS over-activation with higher penetrance and decreased latency as compared to VavP*-Bcl2* tumors alone. Tumors with CTSS alterations were characterized by a remodeled tumor-prone microenvironment with an increased infiltration of CD4^+^ T cells while limiting CD8^+^ T cells recruitment. Depletion of CD4^+^ T cells in *VavP-Bcl2*/CTSS chimera models confirm that CTSS is essential to support the communication and co-stimulatory signals between tumor B cells and CD4^+^ cells in the GC context. Conversely, loss of CTSS activity in aggressive mouse lymphoma xenograft restrain lymphoma growth by recruiting and enhancing CD8^+^ T cells cytotoxic activity while impairing communication with CD4^+^ T_FH_ cells. These data show for the first time that by altering the processing of antigenic peptides, CTSS mutations or overexpression remodel the immune microenvironment to promote lymphoma growth and implies that targeting a regulator of antigen presentation such as CTSS could modulate the spectrum of processed antigens, promote activation of cytotoxic T cells, enhance tumor immunogenicity and improve response to anti-PD1 immunotherapies.

### Disruption of Gα Migration Pathway

Another pathway frequently mutated in GC-derived lymphomas is the GC homing pathway involving *S1PR2* and *GNA13*. The guanine nucleotide binding protein *GNA13* (encoding *Gα13*), is a signaling mediator downstream of transmembrane G-protein-coupled receptors sphingosine-1-phosphate receptor-2 (S1PR2), that confines B cells in the GC and promotes growth regulation by suppressing both Akt and cell migration (110). In about 20% of GCB-DLBCL cases (38% in the EZB subtype), deleterious mutations affect one of the members of the *Gα13* homing pathways, namely *GNA13, S1PR2* and *P2RY8* (another S1P receptor expressed on GC B cells). To model *in vivo* the impact of *GNA13* loss during lymphomagenesis, Muppidi and colleagues utilized a mixed BM chimeric approach, deleting *Gα13* in all B-lineage using *Mb1^Cre^;Gα13^f/f^* mice. Deficiency of *Gα13* favors the formation of enlarged mesenteric LN with GC B cells expansion associated with a marked disruption of the GC architecture and a loss of GC confinement due to the inability to suppress migration in response to S1P. Occasional transformation in B cell lymphomas displaying a GC-like phenotype (Gl7^+^CD138^-^Bcl6^+^Irf4^+^IgD^-^) were observed at 1 year of age (82). One of the critical observations made in *Gα13* deficient cells is the loss of confinement which allows egress outside the GC, dissemination and seeding of these tumoral cells at distant sites such as blood and BM. As deficiency in S1PR2 did not phenocopy *Gα13* deficiency, the authors searched for additional Gα13 G protein coupled receptor that may be involved in GC B cell regulation and discovered that P2RY8, an orphan receptor also represses GC B cell growth promoting confinement *via* Gα13 and is mutated in GCB-DLBCL. Cooperation between Bcl2 overexpression and *Gna13* loss showed an exacerbated phenotype in double mutant mice leading to greater accumulation of GC B cells in spleen, wider dispersal throughout the follicles and more dissemination in blood and BM suggesting a combinatorial effect of Bcl2 in promoting abnormal B cell survival outside the GC niche. These findings shed new lights on an important mechanism by which disruption of Gα13 signaling exerts dual actions in promoting growth and favoring dissemination of GC B cell in GC-derived lymphomagenesis and offer a biological explanation for factors leading to systematic dissemination of tumoral GC B cells in multiple organs including the BM.

## Modeling Dysregulation of GC Metabolism

During T-dependent adaptive immune responses, B cells undergo a quick anabolic shift that sustain the GC proliferative burst (111). Many B-cell lymphomas originating in the GC present an exceptionally high proliferation index. This implies massive metabolic requirements in order to generate sufficient energy and support anabolism for repeated growth and division cycles. Recurrent mutations in components of the nutrient sensing pathway that activates the mechanistic target of rapamycin complex 1 (mTORC1), a driver of cellular anabolism are found in about 25% of FLs (112, 113). In response to growth factors and when there is sufficient intracellular amino acid concentration, mTORC1 promotes protein synthesis (114). Intracellular amino acid concentration is perceived through a protein complex present on lysosome surface comprising RAG GTPases, Ragulator complex, v-ATPase complex (vacuolar H^+^-adenosine triphosphate ATPase) and SLC38A9 (sodium-coupled amino acid transporter 9). When amino acid concentration is sufficient, the RAG GTPases form heterodimers on lysosome surface allowing the recruitment of mTORC1 and downstream protein synthesis supporting cellular growth (114–116).

### *RRAGC* Activating Mutations

The RRAGC gene encodes for Ras-related GTP-binding protein (RAGC), an essential activator of mTORC1 downstream of the sensing of cellular nutrients. Recurrent point mutations of *RRAGC* are found in 18% of FLs and are rare in other B cell lymphomas (112, 113). More than half of these events are associated with mutations of certain components of the v-ATPase complex (*ATP6V1B2* and *ATP6AP1*). To characterize the impact of recurrent *RRAGC* mutations on B cell function and lymphomagenesis, Ortega-Molina et al. used CRISPR/Cas9 genome editing techniques to engineer two independent murine *Rragc* knock-in mouse strains reproducing hotspot mutations recurrently found in human FL (S75C and T90N mutations) (83). Heterozygous RagC^S74C/+^ and RagC^T89N/+^ heterozygous mice showed no obvious phenotype. Lymphoid and myeloid cell populations frequencies were similar in mutant versus wild-type mice in the spleen and BM however RagC knock-in mutations conferred partial insensitivity to nutrient withdrawal. When bred to a VavP-*Bcl2* mice, RagC-Bcl2^+^ double-mutant mice showed exacerbated B cell responses in response to immunization characterized by enlarged GCs, increased plasma cell production without impairment of high-affinity B cell selection and eventually acceleration of lymphoma development. This was observed both in the progeny of *VavP-Bcl2*-RagC^mut^ mice but also using a BM chimeric system where VavP*-Bcl2*-RagC^mut^ fetal liver cells were transplanted into lethally irradiated wild-type recipients. Histological analysis of these tumors revealed a follicular growth pattern of Bcl6^+^ B cells with no difference according to genotype. Bulk RNA sequencing analysis of B220^+^ cells obtained from RagC^mut^ and RagC^WT^ Bcl2^+^ FL-like tumors revealed that the mTORC1 signaling signature was enriched in RagC^mut^ FL. Furthermore, when transcriptional profiles from murine and human FL with or without RagC mutations were compared, upregulated genes in murine RagC^mut^ FL were enriched in *RRAGC* mutated human FL. Interestingly, the mTOR inhibitor rapamycin, given orally to RagC^mut^ and RagC^WT^ Bcl2^+^ mice during long term, inhibited proliferation selectively in RagC^mut^ FL. Overall, this data shows that *RRAGC* mutations result in mTORC1 activation regardless of the intracellular amino acid concentration which confers a selective advantage to GC B cells. In addition, the presence of these mutations makes GC B cells less dependent to T_FH_ signals, as blockade of T cell help through anti-CD40L after GC induction makes RagCmut B cells intrinsically resistant to apoptosis despite T_FH_ suppression. Finally, when these mutations are associated with Bcl2 overexpression, the lymphomagenesis process is accelerated in a cell-intrinsic manner where mutant GC B cells with increased fitness could continue to undergo cycles of selection and proliferation favoring further acquisition of additional hits (83).

### *SESTRIN1* Loss of Function

*SESTRIN1* has been identified as a target gene of the recurrent 6q deletion in FLs and it is also epigenetically inactivated by *EZH2* gain-of-function (84). SESTRIN1 is a mTORC1 regulator that inhibits cell growth when TP53 is activated in response to DNA damage. Inactivation of SESTRIN1, by del6q or by the *EZH2* mutation leads to mTORC1 activation. Therefore, SESTRIN1 loss represents an alternative to *RRAGC* mutations that maintain mTORC1 activity under nutrient starvation. To recapitulate *SESTRIN1* deficiency *in vivo*, in combination with BCL2 overexpression, Oricchio and colleagues retrovirally transduced *VavP-Bcl2* HSPCs with shRNAs targeting *Sestrin1*. *Sestrin1* deficiency led to an acceleration of the Bcl2-mediated lymphoma, establishing his tumor suppressive role in B cells. Morphologically, BCL2/Sestrin1-deficient tumors resembled FL with a follicular architecture, they displayed markers of a GC phenotype (PNA and BCL6), showed evidence of somatic hypermutation, an increased tumor proliferation based on Ki67 staining. Remarkably, pharmacological inhibition of EZH2 promotes SESTRIN1 re-expression and restores its tumor suppressive activity, suggesting the possibility to epigenetically control mTORC1 activity in lymphoma. Interestingly, *EZH2*/*RRAGC* gain of function mutations and *SESTRIN1* loss are mutually exclusive suggesting that these alterations are involved in mTORC1 activity maintenance allowing tumor cells to escape proliferation inhibition (84) also becoming less dependent to T_FH_ signals as shown in the context of *RRAGC* and *EZH2* mutations (83, 101). In summary, mTORC1 pathway activation, through various mutually exclusive molecular alterations, is clearly involved in B cell lymphomagenesis process and may be associated to a specific sensitivity to mTOR inhibitors.

## Conclusions and Perspectives

B cell lymphomas are among the most frequent hematopoietic malignancies and represent a molecularly heterogeneous group of diseases with different therapeutic vulnerabilities (26, 117) and clinical outcomes that largely depend on a complex interplay between a multitude of genomic alterations and heterogeneous tumor microenvironment signatures (118, 119). Sequencing studies allowed the identification of recurrently mutated genes that drive lymphomagenesis and led to refine DLBCL classification that pave the way for personalized therapeutic strategies (14, 19, 118). The latest large-scale genome sequencing studies identified up to seven different molecular subtypes of DLBCL (118) which represent an interesting framework for the development of models mimicking each molecular subtype. Now, recent DLBCL classifications based of TME composition revealed four distinct microenvironment compositions which provide independent contributions to clinical outcome regardless of the GCB/ABC cell-of-origin or the genetic DLBCL subtype classification (119). The challenge of modeling future preclinical mouse models will be to recapitulate both the genomic features of the tumors and the parallel remodeling of the tumor microenvironment. Such tumors likely develop over decades in humans before clinical symptoms appear and rely on a complex combination of hits that have been accumulated in a stepwise and ordered manner leading to a spatially and temporally intra-tumor heterogeneity. Advanced technologies are emerging for a better characterization of the existing and future models and cellular heterogeneity in murine lymphomas can be resolved by measuring gene expression at a single cell resolution, allowing the study of B cells together with their microenvironment. Using those techniques, we and others have started to uncover and compare the underlying transcriptional and functional heterogeneity within FL malignant B cells and normal GC B cells in human and mice (31, 81, 101, 120, 121).

Another important feature of lymphoma biology that need to be properly addressed in mouse models is mimicking the kinetics and cellular context in which genetic hits accumulate overtime sometimes over years. The order of appearance of such hits and how it influences lymphomagenesis remains an area of active research. Mutations in *KMT2D*, *CREBBP* or *EZH2* represent early events during lymphomagenesis and have been proposed to occur in HSPCs. Mouse models reproducing these alterations do not induce lymphomagenesis on their own but accelerate GC B cell transformation when combined with BCL2 overexpression (68–71). Accordingly, recent genomic data obtained from prediagnostic samples formally demonstrate that BCL2 translocations systematically precede the acquisition of subsequent epigenetic mutations (122).

Murine models developed since several decades have been particularly useful to inform us on the biological mechanisms driving B cell lymphomagenesis, and helping to understand the functional consequences of genetic alterations. As one of the emerging functions of epigenetic modifier genes in GC B cell lymphoma is to favor disease initiation through the reprogramming of the immune niche, mouse models that faithfully recapitulate the complex interactions with the microenvironment will become valuable tools for the testing and development of novel, rationally designed therapeutic approaches (80, 101). In this line, it is remarkable that microenvironment remodeling seems to occur early in the context of *EZH2* mutations, suggesting that early epigenetic therapy may be useful to prevent disease progression (105).

GEM models may recapitulate the natural history and histological properties of human tumors. However, they display a limited mutational and immunological complexity compared to the human tumors they are intended to model (119). Novel sophisticated tools for engineering mouse models such as CRISPR-Cas9 gene editing techniques or patient-derived xenografts (especially those mimicking indolent lymphomas such as FL), including in humanized mice, will be useful to fill this gap and efficiently generate preclinical models that reflect the complex genetics of the human tumors (61, 123–125). The generation of transplantable lymphoma cell lines obtained from these GEM can be engineered to develop functional CRISPR screening *in vivo;* such an approach would also be useful to screen for therapeutic vulnerabilities in an unbiased manner. Now that most of the genetic drivers have been discovered in B cell lymphomas and that we are getting the tools for a more in-depth characterization of their functional consequences both on B cells and its microenvironment, we hope that studies using more complex GEM tumor models will serve to streamline the translation of targeted therapies with novel immunotherapies into the clinics.

## Author Contributions

All authors contribute to the writing of the review. All authors contributed to the article and approved the submitted version.

## Funding

This work was supported by grants from Fondation pour La Recherche Médicale (GB), Fondation ARC, Institut National du Cancer, Canceropôle PACA and Institut Carnot Calym. We thank all the team members past and present that have contributed to some of the work described in this review.

## Conflict of Interest

The authors declare that the research was conducted in the absence of any commercial or financial relationships that could be construed as a potential conflict of interest.
